# Individual differences in the expansiveness of mental disorder concepts: development and validation of concept breadth scales

**DOI:** 10.1186/s12888-023-05152-6

**Published:** 2023-10-04

**Authors:** Jesse S. Y. Tse, Nick Haslam

**Affiliations:** Melbourne School of Psychological Sciences, University of Melbourne, Victoria, 3010 Australia

**Keywords:** Concept breadth, Concept creep, Mental disorder, Mental illness, Mental health literacy, Stigma, Help-seeking attitudes

## Abstract

**Background:**

What people consider to be a mental disorder is likely to influence how they perceive others who are experiencing problems and whether they seek help for their own problems. However, no measure is available to assess individual differences in the expansiveness or breadth of concepts of mental disorder. Four studies aimed to develop and validate two such measures. The Concept Breadth-Vertical (CB-V) scale assesses variability in the severity threshold at which unusual behavior or experience is judged to reflect disorder, whereas the Concept Breadth-Horizontal (CB-H) scale assesses variability in the range of phenomena judged to be disorders.

**Methods:**

In a pilot study (*N* = 201) for the CB-V, participants read vignettes of varying severity for each of the 10 mental disorders, and rated whether the subject had a disorder. Study 1 (*N* = 502) used exploratory factor analyses to examine 10 CB-V items from the pilot study and 20 vignette-based items for constructing the CB-H. Study 2 (*N* = 298) employed confirmatory factor analysis to validate the scales’ structure and examined their convergent validity with a measure of harm concept breadth and their discriminant validity with measures of mental health literacy. Study 3 (*N* = 298) explored associations of the scales with other mental health variables, including stigma and help-seeking attitudes.

**Results:**

Study 1 supported the unifactorial structure of each item set, refined each set into a scale, and demonstrated acceptable reliabilities. Study 2 provided support for the scales’ convergent and discriminant validities. Study 3 showed that the scales were associated negatively with stigma, and positively with help-seeking attitudes and self-reported mental health problems. Studies 2 and 3 further indicated that younger and more politically liberal participants hold broader concepts of mental disorder.

**Conclusions:**

The new concept breadth scales are psychometrically sound measures of a promising new concept in the study of beliefs and attitudes about mental health. Potential future research directions are discussed.

**Supplementary Information:**

The online version contains supplementary material available at 10.1186/s12888-023-05152-6.

## Background

How “mental disorder” should be defined and delimited has been a topic of philosophical and clinical debates for many decades [1]. Theorists have proposed abstract definitions or deny that any clear-cut definition is possible (e.g., [2, 3]). Psychiatric classifications provide lists of officially recognized disorders that serve as “ostensive” definitions of what the concept of disorder includes. When these classifications are revised, critics argue about the inclusion or exclusion of particular disorders and shifts in the diagnostic criteria for particular disorders (e.g., [4–7]). These disputes hinge on where the boundary between disorder and non-disorder should be drawn. The placement of that boundary has significant implications for clinical practice, for research, and for the people whom it includes or excludes.

Discussions over the definition of mental disorder often focus on the expansiveness of the boundary. Recurring arguments over the expansion of the concept are often framed in terms of “diagnostic inflation” [8], “psychiatrization” [9], “medicalization” [10, 11], “pathologization” [12], or “disease mongering” [13], reflecting a concern that psychiatric classifications have broadened the range of psychological phenomena that count as disorders [14, 15]. Critics of this supposed expansion contend that it promotes over-diagnosis along with unnecessary and potentially harmful treatment, as well as endangering our sense of normality [16–18]. Advocates of expansion often counter-argue that new diagnoses or increasingly expansive criteria for existing disorders can identify people in genuine need of clinical attention [19, 20].

Most of the debate over the expansiveness or breadth of definitions of mental disorder has focused on philosophical definitions and official classifications. However, it is equally important to understand how laypeople define mental disorders. Lay concepts of mental disorder are more likely to influence how members of the public perceive people experiencing mental illness and whether or not they seek help for their problems than concepts advocated by philosophers and psychiatric nosologists. Several lines of research have examined laypeople’s “illness beliefs” about specific disorders and the idioms of distress that are prominent in their cultures (e.g., [21, 22]), but relatively little research has quantitatively examined how laypeople define mental disorder as a general concept. Several studies (e.g., [23, 24]) have explored this question and examined cultural differences in the breadth and key defining features of disorder concepts. For instance, Tse and Haslam [25] found that American participants tended to hold a concept of disorder that was similar in breadth to the *DSM-5* but not closely aligned with it, and that their disorder judgments were primarily based on the extent to which a person’s problems involved severe harm (distress and impairment) and were rare.

One novel approach to this topic has been developed in theory and research on “concept creep”. Haslam [26] proposed that in recent decades many concepts related to harm have undergone a semantic expansion, so they now refer to a broader range of phenomena than previously. For example, in psychology “bullying” initially referred to intentional, repeated aggression perpetrated downwards in a power hierarchy among children, but it has gradually expanded its reach so now it may refer to unintentional, unrepeated aggression that is commonly perpetrated laterally or even upward in adult workplaces. Haslam and colleagues have argued that concept creep is driven by a rising cultural sensitivity to harm and takes two forms. Horizontal creep occurs when a concept broadens to include qualitatively new phenomena, such as when “bullying” is applied to adults rather than only to children, or when “addiction” is expanded to encompass compulsive behaviors that do not involve ingestion of substances, such as gambling. Vertical creep occurs when a concept broadens to include quantitatively less severe phenomena, such as when bullying expands to include unrepeated behavior or “addiction” includes problematic substance use without physiological dependency.

The theory of concept creep draws attention to the breadth of concepts as a focus of research attention. This focus on “concept breadth” – the semantic range that increases when concept creep takes place – can be applied to both professional and lay concepts and examined in relation to any harm-related concept, including mental disorder. In principle, the breadth of people’s concepts of disorder can be measured and the causes, correlates, and consequences of individual or group differences in concept breadth can be investigated. Individual difference measures of the breadth of several harm-related concepts have been developed and found to be associated with an assortment of demographic, personality, and attitudinal variables [27, 28]. Developing a measure specific to the concept of mental disorder would enable a program of research into the implications of broad versus narrow lay concepts of mental disorder that might complement and illuminate theoretical discussions of how mental disorder should be defined and of the implications of diagnostic inflation.

The range of mental health-related phenomena that mental disorder concept breadth might be associated with is potentially large, but stigma and help-seeking are two promising candidates. Stigma refers to stereotypes, prejudice, and discrimination towards people with mental disorders [29], including perceptions that they are dangerous and unpredictable and a tendency to seek social distance from them. A vast body of research has examined the predictors of stigmatizing attitudes and documented the negative implications they have for the well-being of people experiencing mental ill-health and their likelihood of seeking treatment (e.g., [30–33]). Although little or no research has examined the possibility, people holding broader concepts of mental disorder might have less stigmatizing attitudes because they are more likely to see mental disorder as common and relatively “normal” rather than rare and deviant. If stigma partly reflects fear or disapproval of social deviancy, a vertically and horizontally broad concept of mental disorder should undermine it.

Broader concepts of mental disorder may also promote help-seeking for mental health problems. Although stigma is one well-established factor discouraging people from seeking help, another plausible factor is holding a narrow concept of mental disorder. People holding such a concept may be less likely than others to identify their experiences as a case of mental disorder and therefore less likely to see professional help as appropriate. Preliminary evidence for this possibility was provided by Tse and Haslam’s [34] study, which found that Americans with a broader concept of mental disorder held more positive attitudes toward help-seeking, and that the larger concept breadth of White Americans relative to Asian Americans partially accounted for their more positive help-seeking attitudes. It remains to be determined whether mental disorder concept breadth is associated with actual help-seeking behavior in addition to help-seeking attitudes and whether it clarifies other cultural or ethnic differences. Similarly, whether individual differences in concept breadth are related to other mental health-related phenomena – the likelihood of self-diagnosis, the risk of developing disorders, the belief that mental disorder falls on a continuum with normality rather than being categorically separate, the preference for certain explanatory models of mental disorder, and so on – awaits further research. Research on any of these relationships requires the development of a validated measure of mental disorder concept breadth.

Any attempt to validate such a measure and advance a program of research on concept breadth must evaluate its relationship to related constructs. One key construct is mental health literacy, the accurate understanding and knowledge of mental disorders and their treatments [35]. Greater mental health literacy – which is associated with being female [36–38], more educated [37–39], and higher socioeconomic status (SES; [40]) – has been found to predict better recognition of signs and symptoms and more professional help-seeking [41]. Interventions that target mental health literacy have been shown to be effective in decreasing stigma [41] and improving other mental health outcomes [42, 43].

Although the constructs of mental disorder concept breadth and mental health literacy have superficial similarities, they are conceptually distinct. Mental health literacy relates to the factual accuracy of knowledge, based on correspondence with expert knowledge in the mental health professions (e.g., the DSM classification; [44]), whereas mental disorder concept breadth relates not to accuracy but to the expansiveness of people’s beliefs of what counts as a disorder. A person could hold a broad but inaccurate concept, a narrow but accurate concept (if at least most recognized mental disorders were correctly identified as such), and any other combination. In principle, breadth and accuracy are separate features of people’s concepts of mental disorder. However, determining whether they are relatively independent in practice awaits an adequate measure of mental disorder concept breadth, and it will be important to establish whether any links between concept breadth and other mental health-related variables, such as stigma, are not attributable to mental health literacy.

**Fig. 1:**
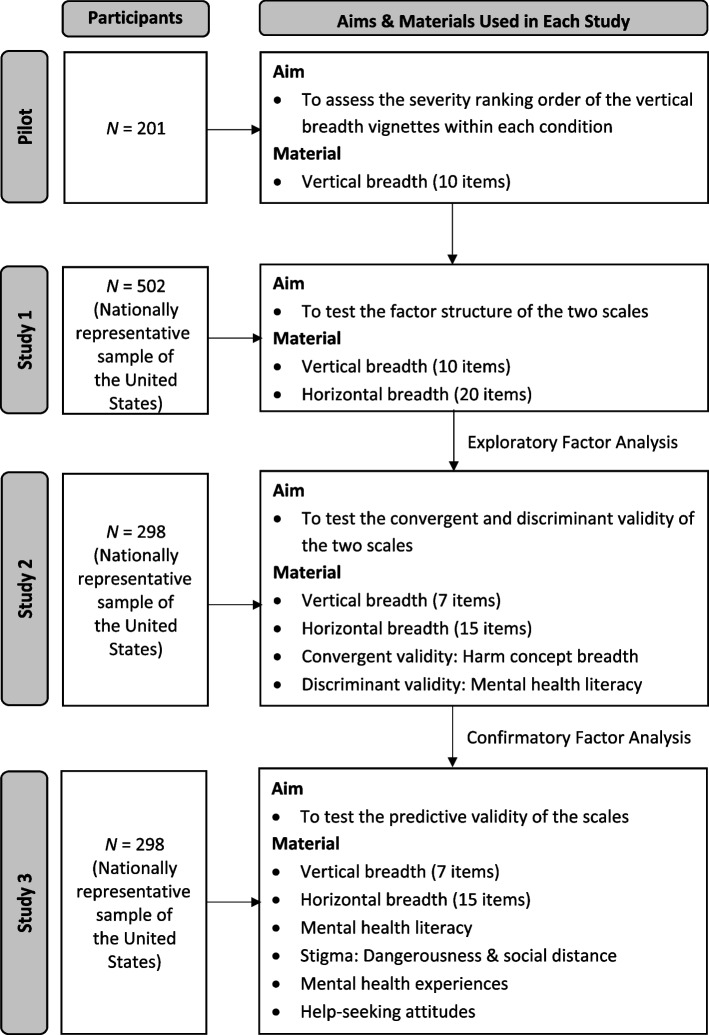
Overview of studies for the development of two scales

The present research includes a series of studies that aimed to develop and validate new measures of mental disorder concept breadth that would capture for the first time the vertical and horizontal dimensions of concept breadth. The validation process aimed to evaluate the factor structure of the measures, their discriminant validity vis-à-vis mental health literacy, and their capacity to predict prominent mental health-related variables, notably stigma, help-seeking, and personal experience with mental disorder. An overview of the studies is presented in Fig. 1. The overarching goal of the research was to develop psychometrically robust scales to enable future research on a novel and promising construct.

## Study 1

The first study in the series aimed to develop sets of items for the two proposed scales, refine these sets, and carry out a preliminary analysis of their latent structure. A pilot study developed candidate items for the vertical breadth scale (CB-V), and the main study used exploratory factor analysis of the respective item sets to test for a unifactorial structure in each and to identify and remove weak items.

### Method

#### Participants

Following recommendations that a sample of 500 is very good for scale development purposes [45] and that the participant-to-variables ratio should exceed 10 in factor-analytic research [46], we recruited 536 participants from Prolific Academic who were paid for their participation. The sample was nationally representative of the United States of America, stratified by gender, age, and race. Of the 536 participants, 34 were excluded due to failing two out of three attention checks and/or not following instructions. The data analysis was conducted on 502 participants. Demographic characteristics for this study and Studies 2 and 3 are presented in Table 1.

**Table 1:** Demographic composition of studies 1 – 3

	Study 1 (*N* = 502)	Study 2 (*N* = 298)	Study 3 (*N* = 298)
Age			
Range	18–84	18–85	18–91
Mean (*SD*)	45.00 (15.85)	45.76 (16.19)	44.20 (15.76)
Gender			
Men	241	140	138
Women	253	155	152
Other	8	3	8
Ethnicity			
White	357	213	219
Hispanic or Latino/a	30	19	17
Black or African American	69	40	37
Asian	37	17	18
Other	9	9	7
Education			
Some high school	6	2	3
High school graduate	61	40	32
Some college	114	64	72
Associate degree	55	37	27
Bachelor's degree	179	109	110
Master's degree	64	41	46
Doctoral degree	23	5	8
Annual income (USD)			
Less than $50,000	273	188	161
$50,000–99999	156	78	93
$100,000–149999	39	17	27
$150,000 or more	21	6	10
Prefer not to say	13	9	7

#### Materials

##### Vertical scale items

Ten vignettes, each describing a *DSM-5* disorder, were selected from a set of 61 vignettes developed by Tse and Haslam [25]. The 10 diverse disorders – schizophrenia, bipolar I disorder (manic episode), major depressive disorder, generalised anxiety disorder, obsessive compulsive disorder, dissociative identity disorder, binge eating disorder, conduct disorder, gambling disorder, and avoidant personality disorder – were chosen for their relatively high familiarity to the public. Based on each original vignette’s mean rating on the item “This person has a mental disorder” from Tse and Haslam’s [25] study, four new versions of each vignette were written to describe varying levels of severity of the same disorder both below and above the original version. The intended outcome was a set of five vignettes for each disorder whose severity levels increased in small steps from a clearly subthreshold case to a case that clearly exceeded the diagnostic threshold. Severity variations were carefully calibrated by varying the intensity, duration, impairment and/or number of symptoms presented in the vignette, although all other aspects of the vignettes were held constant across all five versions.

We conducted a pilot study (*N* = 201) on the 10 vertical breadth items to assess the severity ranking order of the vignettes within each condition. The main focus of the pilot study was to ensure a relatively uniform structure of these 10 sets of vignettes by having approximately equal intervals in rated severity between each severity level. A sample of 204 Americans was recruited via Prolific. Three participants were excluded for failing at least two out of the three attention check questions. The final sample of 201 had a mean age of 35.66 (*SD* = 14.48); the majority were white (69.70%) and approximately half were men (50.70%). After posting an advertisement on Prolific, interested platform users were directed to a Qualtrics link where they provided consent before being randomly allocated to one of the two groups (*n*s = 100 & 101). Participants in each group rated 25 vignettes – all five severity levels for a different set of five disorders, depending on the group – on the item “This person has a mental disorder” (1 = *Strongly disagree* to 6 = *Strongly agree*). We inspected the mean ratings to ensure they followed the intended severity rank order, and they did for all 10 disorders. Minor adjustments to vignette wording were then made in an effort to equalize the rating interval between successive severity levels (e.g., subtly decreasing the severity of a vignette if its mean rating was too close to that of the vignette at the next more severe level). The pilot study therefore resulted in a revised set of 50 vignettes describing five severity levels of 10 *DSM-5* disorders.

Study 1 therefore contained 10 candidate items for a vertical concept breadth scale, each containing five vignettes varying in level of severity. Each item was presented on a single page from most severe (top) to least severe (bottom) with a question prompt at the top “Do any of these people described below have a mental disorder?”. For each vignette, participants judged whether the person described in it has a mental disorder with a “Yes” or “No” response. Each item was then scored from 0 to 5 based on the number of “Yes” responses. Higher scores indicate greater vertical breadth or a lower disorder judgment threshold.

##### Horizontal scale items

As with the candidate vertical scale items, the candidate items for the horizontal breadth scale were sourced from Tse and Haslam’s [25] vignettes. Ten *DSM-5* disorders (persistent depressive disorder, social anxiety disorder, posttraumatic stress disorder, somatic symptom disorder, insomnia disorder, gender dysphoria, delirium, mild neurocognitive disorder, narcissistic personality disorder, and sexual masochism disorder) and 10 non-disorders (recurrent cheating, jealousy, selfishness, poor hygiene, social media disorder, chronic fatigue syndrome, prosopagnosia, internet gaming disorder, Dhat, and imposter syndrome) were selected. These vignettes were chosen based on three considerations. First, they did not overlap with those used for the vertical scale candidate items. Second, a substantial set of *DSM-5* non-disorders was sampled because having a broad concept of mental disorder may entail judging conditions to be disorders beyond official psychiatry’s current boundary (false positives). Third, we aimed to select vignettes that would elicit varying judgments from participants rather than high levels of consensus. Thus, vignettes were selected based on having mean ratings on the item “This person has a mental disorder” close to the 3.5 mid-point (on a 6-point Likert scale) and a relatively large standard deviation (*SD* > 1.30) in Tse and Haslam’s [25] study. The candidate items therefore had roughly equal numbers of participants judging them to be disorders or not to be disorders.

In Study 1, participants rated the 20 candidate horizontal breadth scale items on their agreement with the statement “This person has a mental disorder” on a 6-point Likert scale (1 = *Strongly disagree* to 6 = *Strongly agree*).

#### Procedure

This research project was approved by the Human Research Ethics Committee of the University of Melbourne. Prolific users were directed to a Plain Language Statement and gave their consent prior to participating in the survey on Qualtrics. They completed the candidate vertical breadth items followed by the candidate horizontal breadth items, both sets in randomized order for each participant, and then responded to demographic questions (age, gender, ethnicity, education, income, political orientation, years living in the United States, first language, and English proficiency), before being debriefed and paid.

### Results

#### Vertical breadth items

The suitability of the data for exploratory factor analysis was reflected by a high value of Kaiser–Meyer–Olkin measure of sampling adequacy of 0.89 and a significant Bartlett’s Test of Sphericity, *χ*^*2*^ (45) = 1181.40, *p* < 0.001. The parallel, MAP, and scree tests all suggested a one-factor solution. Using the maximum likelihood method, the model accounted for 38.94% of the variance. Factor loadings are presented in Table 2. The three lowest-loading items (the conduct disorder, schizophrenia, and gambling disorder vignette sets), which also had relatively low communalities (0.21 to 0.27), were removed for the final vertical breadth scale (CB-V). The remaining seven items showed very good internal consistency (Cronbach’s alpha = 0.82). For detailed wordings of the retained vertical breadth items, see Additional file 1: Appendix A.

**Table 2:** Mean item scores and factor loadings for the vertical breadth candidate items

Item	Mean	*SD*	Factor loadings
Generalized anxiety disorder	3.01	1.43	.69
Major depressive disorder	3.29	1.57	.63
Bipolar I disorder	3.05	1.65	.62
Avoidant personality disorder	2.82	1.76	.60
Binge eating disorder	2.80	1.60	.57
Obsessive compulsive disorder	3.65	1.13	.53
Dissociative identity disorder	3.48	1.20	.53
Conduct disorder ^a^	3.54	1.50	.52
Schizophrenia ^a^	3.10	0.93	.49
Gambling disorder ^a^	3.33	1.93	.46

^a^Item was removed for the final vertical scale (CB-V)

##### Vertical breadth – short form (CB-V-S)

In light of the complexity and time required to read and judge the 35 vignettes (seven items, each with five severity levels) in the CB-V scale, we created a short form. We selected the most marginal vignette for each of the 10 original items (i.e., the vignette closest to an even split of participants answering “Yes” and “No” to the statement “This person has a mental disorder”) and these made up the CB-V-S (see Additional file 1: Appendix B). The CB-V-S therefore includes 10 threshold items with a dichotomous “Yes” (1 score) or “No” (0 score) response (possible score range 0 – 10). Based on Study 1 data, we conducted a principal component analysis (PCA) to evaluate the unidimensionality of the short form. Parallel, MAP, and scree tests all suggested a one-component solution, which accounted for 32.81% of the variance. Component loadings are presented in Table 3. The CB-V-S also had a good internal consistency with a Cronbach’s alpha of 0.77 and correlated 0.87 (*p* < 0.001) with the CB-V, indicating a very strong convergence.

**Table 3:** Mean item scores and component loadings for the vertical breadth – short form (CB-V-S)

Item	Mean	*SD*	Component loadings
Major depressive disorder	0.53	0.50	.61
Generalized anxiety disorder	0.33	0.47	.61
Bipolar I disorder	0.43	0.50	.60
Binge eating disorder	0.56	0.50	.60
Obsessive compulsive disorder	0.61	0.49	.57
Gambling disorder	0.45	0.50	.57
Dissociative identity disorder	0.51	0.50	.56
Conduct disorder	0.57	0.50	.56
Avoidant personality disorder	0.58	0.49	.55
Schizophrenia	0.26	0.44	.49

#### Horizontal breadth items

The suitability of the data for exploratory factor analysis was supported by a high value of Kaiser–Meyer–Olkin measure of sampling adequacy of 0.86 and a significant Bartlett’s Test of Sphericity, *χ*^*2*^ (190) = 2161.03, *p* < 0.001. The parallel test suggested a three-factor solution while the MAP and scree tests suggested a one-factor solution, which was followed. Using the maximum likelihood method, the model accounted for 25.05% of the variance. Factor loadings are presented in Table 4. Five items were removed based on having the lowest factor loadings (0.15 to 0.32) and communalities (0.02 to 0.10). The reliability of the remaining 15 items was very good (Cronbach’s alpha = 0.83). Reassuringly, all retained items had mean ratings near the midpoint of the disorder judgment scale and high response variability. For detailed wordings of the retained horizontal breadth items, see Additional file 1: Appendix C.

**Table 4:** Mean item scores and factor loadings for the horizontal breadth candidate items

Item	Mean	*SD*	Factor loadings
Imposter syndrome	3.09	1.32	.59
Social media disorder	3.57	1.34	.58
Narcissistic personality disorder	4.02	1.53	.57
Jealousy	3.64	1.31	.57
Internet gaming disorder	4.18	1.37	.56
Chronic fatigue syndrome	3.14	1.38	.53
Insomnia disorder	3.13	1.37	.52
Social anxiety disorder	2.77	1.31	.50
Recurrent cheating	2.62	1.30	.50
Selfishness	3.43	1.56	.50
Posttraumatic stress disorder	4.07	1.49	.46
Dhat	3.76	1.44	.46
Persistent depressive disorder	4.13	1.26	.45
Somatic symptom disorder	3.16	1.40	.44
Delirium	3.58	1.41	.39
Poor hygiene ^a^	3.06	1.41	.32
Mild neurocognitive disorder ^a^	3.45	1.36	.30
Sexual masochism disorder ^a^	3.49	1.53	.29
Prosopagnosia ^a^	4.24	1.38	.22
Gender dysphoria ^a^	2.35	1.36	.15

^a^Item was removed for the final horizontal scale

### Discussion

Study 1, including its pilot study, represents a thorough and systematic scale development process that employed an extensive set of vignettes. Preliminary evidence supports the unifactorial structure of the two new scales, and after the elimination of psychometrically weaker items, the scales appear to meet very good standards of internal consistency. The CB-H scale is a relatively typical vignette-based measure whose items appear to successfully capture relatively marginal examples of DSM-5 conditions that roughly half of our participants judged to be, or not to be, mental disorders. The CB-V scale is a more unusual scale that uses severity-ranked sets of vignettes to identify participants’ thresholds for judging where “normality” ends and disorder begins, akin to a psychophysical task for assessing perceptual thresholds. This innovative scale format yields adequate reliability and may serve as a valuable supplement to the measure of horizontal concept breadth. A more conventional short version also offers reliable measurement.

## Study 2

Study 2 used the CB-H and CB-V scales and had four primary goals. First, it aimed to check the reliability of the scales in a new sample. Second, it used confirmatory factor analysis to conduct a stronger test of the unifactorial structure of the two scales. Third, the study began the process of validating the new scales by testing whether they converged with an established concept breadth scale and diverged from measures of mental health literacy. The latter construct might be superficially confused with concept breadth but relates to the accuracy of mental health knowledge rather than the breadth of the concept of what counts as a mental disorder, and there is little or no reason a priori why greater literacy should covary with greater or lesser concept breadth. Finally, we aimed to explore possible demographic correlates of concept breadth, noting previous findings suggesting that younger and more politically liberal people tend to hold broader harm concepts [27, 28]. We predicted that both concept breadth scales would be unifactorial, would correlate strongly with the prior measure of concept breadth (although that measure does not include an assessment of vertical breadth), and would correlate weakly or not at all with two measures of mental health literacy.

### Method

#### Participants

We sought a sample of at least 300 participants in view of our plan to conduct confirmatory factor analysis with up to 15 items and to have sufficient statistical power to detect potentially small associations between our scales and participants’ demographic characteristics. Another nationally representative sample of the United States was recruited on the Prolific platform. Out of the 310 complete responses, 12 participants were excluded for the following reasons: not following instructions (5), failing two or more attention checks (5), and straight-line responses (2). The final sample for analysis consisted of 298 participants whose demographic characteristics are presented in Table 1.

#### Materials

##### Concept Breadth – CB-V and CB-H

The seven and 15 items retained from Study 1 for the respective scales were used in the present study. Instructions for participants and scoring were identical to Study 1.

##### Harm Concept Breadth Scale (HCBS) – mental disorder subscale [27]

The HCBS measures the breadth of harm-related concepts, specifically the concepts of bullying, trauma, prejudice, and mental disorder, which are included as four subscales. The mental disorder subscale was employed to test the convergent validity of the newly developed scales. The 10-item subscale assesses individual differences in the breadth of the concept of mental disorder. Participants read 10 vignettes of 30 to 50 words, each describing a person’s experience. They then rated their agreement to “I believe this is an example of mental disorder” on a 6-point Likert scale (1 = *Strongly disagree* to 6 = *Strongly agree*). A higher score indicates greater breadth.

##### Mental health literacy

There is no consensus on the definition or measurement of mental health literacy [47]. Some measures assess one aspect of the construct while others assess multiple aspects. Therefore, two popular measures of mental health literacy were used to test the discriminant validity of the concept breadth scales.

##### Mental health literacy measure [48]

This measure contains 26 items measuring three dimensions of mental health literacy: knowledge-oriented, beliefs-oriented, and resource-oriented mental health literacy. Participants responded with their agreement to the items measuring the first two dimensions on a 5-point Likert scale (1 = *Strongly disagree* to 5 = *Strongly agree*) with the option of “I don't know”. Those who responded “strongly agree” or “agree” were awarded 1 point. The response options for the five items on the resource-oriented dimension were dichotomous “Yes” (1 point) and “No” (0 points). Example items are “Counseling is a helpful treatment for depression” (Knowledge-oriented); “Mental illness is a short-term disorder” (Beliefs-oriented); and “I know where to go to receive mental health services” (Resource-oriented). Scores from the three dimensions were summed with a higher score indicating higher literacy.

##### Mental Health Literacy Assessment for College Students (MHLA-c; [49])

The MHLA-c is a unidimensional measure with some items adapted from the Multiple-Choice Knowledge of Mental Illnesses Test (MC-KOMIT; [50]). There are 18 multiple-choice questions, each with one correct option from five options. For example, “Which of the following is the most common long-term course of dementia?” with options: (a) improvement; (b) paralysis; (c) progression; (d) remission; and (e) stabilization. Participants get 1 point if they answer a question correctly. A higher total score indicates greater literacy. There are three versions of this measure, and form B was used in this study.

#### Procedure

Similar Prolific recruitment and consent processes described in Study 1 preceded the Study 2 survey. Participants then completed a battery of all measures in a randomized order, followed by the same set of demographic questions as in Study 1. Participants were then debriefed and paid for their time.

### Results

#### Associations with demographic variables

Mean scores on the concept breadth scales did not differ by gender – CB-V, *t*(293) = 0.65, *p* = 0.517, and CB-H, *t*(293) = 1.67, *p* = 0.097 – nor by race, dichotomized as White or non-White participants due to the low numbers in most minority groups – CB-V, *t*(296) = 1.36, *p* = 0.174, and CB-H, *t*(296) = 1.80, *p* = 0.072. Age was not significantly associated with the CB-V (*r* = -0.004, *p* = 0.941) nor CB-H (*r* = -0.11, *p* = 0.050). The scales also did not differ according to education level (coded as less than college, some college or Bachelor’s degree, and more than Bachelor’s degree) – CB-V, *F*(2, 295) = 0.67, *p* = 0.514, and CB-H, *F*(2, 295) = 0.38, *p* = 0.683 – nor income — CB-V, *F*(3, 285) = 0.88, *p* = 0.454, and CB-H, *F*(3, 285) = 1.33, *p* = 0.265. CB-H (*r* = -0.17, *p* = 0.004) but not CB-V (*r* = -0.08, *p* = 0.188) correlated with political orientation, indicating that more liberal participants identified a wider range of vignettes as examples of mental disorder than more conservative participants.

#### Confirmatory factor analysis

Confirmatory factor analyses were carried out for the CB-V and the CB-H separately and model fit indices were examined for each. According to Hu and Bentler [51], the comparative fit index (CFI) and Tucker-Lewis index (TLI) > 0.95, and root mean square error of approximation (RMSEA) < 0.06 indicate a good model fit. For the CB-V, the CFI was 1.00, TLI was 1.00, and RMSEA was 0.00, 95%CI [0.00,0.06]. For the CB-H scale, the CFI was 0.95, TLI was 0.93, and RMSEA was 0.05, 95%CI [0.04,0.06]. With the marginal exception of TLI for CB-H, all other indices reflected a good model fit. Cronbach’s alpha for the CB-V and CB-H scales were 0.79 and 0.82, respectively, and the scales correlated positively, *r* = 0.48, *p* < 0.001.

#### Convergent and discriminant validity

The HCBS mental disorder subscale correlated 0.49 (*p* < 0.001) with the CB-V scale and 0.61 (*p* < 0.001) with the CB-H scale. The stronger convergence with the CB-H scale was compatible with the HCBS’s focus on horizontal concept breadth. The CB-V and CB-H scales correlated weakly but positively with the Mental Health Literacy Measure, *r* = 0.19, *p* < 0.001, and *r* = 0.17, *p* = 0.003 respectively, and also with the MHLA-c, *r* = 0.19, *p* < 0.001, and *r* = 0.22, *p* < 0.001, respectively. The modest magnitude of these correlations supported the conceptual and empirical distinctness of concept breadth from mental health literacy.

### Discussion

Study 2 confirmed that the two concept breadth scales represent unitary constructs and can be measured reliably. The study further documents that the scales converge as expected with an existing measure of concept breadth. There are three good reasons to argue that the CB-H and CB-V are likely to be superior measures of concept breadth. First, they were developed through a more thorough scale development process. Second, they were constructed with a specific goal of assessing the breadth of the concept of mental disorder, whereas the HCBS subscale was designed as one element of the broader construct of harm-related concept breadth. Third, the new measures assess both dimensions of concept breadth whereas the earlier measure only assessed the horizontal component. The new scales should therefore be the preferred measure for researchers with a specific mental health-related focus.

Evidence that the new scales are not redundant with mental health literacy, measured using two distinct scales, supports the distinctness of the construct of concept breadth. It suggests that concept breadth may have a unique capacity to predict and explain mental health-related phenomena independently of that well-studied and fruitful construct. Although it might have transpired that people with broad concepts of mental disorder have high levels of literacy – a finding that would be expected if people lacking knowledge tended to have narrow concepts of disorder and were unaware of the range of disorders – that correlation was weak. The most plausible interpretation of that weak association is that people’s beliefs about the range and severity threshold for mental disorders are not strongly linked to their levels of factual knowledge about mental disorder.

## Study 3

Study 3 extended the validation of the concept breadth scales by investigating their associations with several important mental health-related variables. In particular, it examined whether holding broad concepts of mental disorder is associated with stigma towards affected people, with more positive attitudes to help-seeking for mental health problems, and with personal experience of mental ill health. We included a measure of mental health literacy in this study to determine whether an association between concept breadth and other variables are independent of an established predictor of those variables.

We predicted that broad concepts of mental disorder would be associated with less stigmatizing attitudes because such concepts should be linked to perceiving mental disorder as common and normal rather than rare and aberrant. We predicted that broad concepts would also be associated with more positive help-seeking attitudes, consistent with the findings of Tse and Haslam [34], for the same reasons. We had no predictions about associations of concept breadth with personal experience of mental health problems, but positive associations are plausible both because experiencing these problems could expand people’s understanding of mental disorder and because people with broader concepts may be more likely to identify their problems as disorders. In addition to examining these associations, we again explored associations between concept breadth and demographic variables.

### Method

#### Participants

Another nationally representative sample of the United States was recruited on the Prolific platform. Out of the 306 complete responses, eight participants were excluded for not following instructions (7) or failing two or more attention checks (1). The final sample for analysis consisted of 298 participants. The demographic characteristics of the participants are presented in Table 1.

#### Materials

In addition to the two concept breadth scales and the demographic questions, several additional measures were included in the survey.

##### Mental health literacy

The MHLA-c [49] was used to measure individuals’ level of mental health literacy with multiple-choice questions. A description of the scale is presented in Study 2.

##### Stigma

Aspects of stigma were assessed by two well-known scales. To assess perceived dangerousness, we used the Dangerousness Scale [52], in which participants rate their agreement on a 6-point scale (0 = *Strongly disagree* to 5 = *Strongly agree*) to 8 statements such as “If I know a person has been a mental patient, I will be less likely to trust him.” To assess desired social distance, the Social Distance Scale (SDS) was adapted from Link et al. [52]. Its seven questions (e.g., “How would you feel having someone with a mental disorder as a neighbor?”) ask participants about their willingness to interact with a person with a mental disorder in various social contexts. Participants rated their willingness on a 4-point scale (0 = *Definitely willing* to 3 = *Definitely unwilling*). Higher scores on both scales indicate greater stigma.

##### Mental health experience

Four items were written to measure whether participants had experienced any psychological problems previously, whether they had sought professional help, and whether their family or friends had experienced any psychological problems. Participants responded to each of these items with a dichotomous “Yes” or “No” response.

##### Help-seeking attitudes

These attitudes were measured using the Inventory of Attitudes Toward Seeking Mental Health Services (IASMHS) from Mackenzie et al. [53]. This inventory revised the popular measure Attitudes Toward Seeking Professional Psychological Help scale (ATSPPH; [54]), which had been criticized for its limitations on validity [55, 56]. The IASMHS asked participants to rate their agreement with 24 statements (e.g., “It is probably best not to know everything about oneself”) on a 5-point scale (0 = *Disagree* to 4 = *Agree*). The scale has three subscales – “psychological openness”, “help-seeking propensity”, and “indifference to stigma” – but the last was omitted from the present study due to its conceptual overlap with the stigma measures. The IASMHS has a high internal consistency of 0.86 and a test–retest reliability of 0.73 [53].

#### Procedure

Similar Prolific recruitment and consent processes described in Study 1 and 2 preceded the Study 3 survey. Participants then completed a battery of measures in a randomized order, including the concept breadth scales, mental health literacy, perceived dangerousness, desired social distance, mental health experience, and help-seeking attitudes. The same set of demographic questions was asked at the end of the survey. Participants were then debriefed and paid for their time.

### Results

Cronbach’s alpha values for the CB-V and CB-H were 0.78 and 0.82, respectively, similar to the previous studies. Associations between the scales and most demographic variables were generally consistent with those obtained in Study 2. There were no significant differences by gender – CB-V, *t*(288) = 0.89, *p* = 0.377, CB-H, *t*(288) = 0.55, *p* = 0.586 – by race (White vs non-White) – CB-V (*t*(296) = -1.44, *p* = 0.151), CB-H (*t*(296) = -1.73, *p* = 0.085 – or by education level – CB-V, *F*(2, 295) = 1.86, *p* = 0.157, CB-H, *F*(2, 295) = 0.86, *p* = 0.423. As in Study 2, more liberal participants tended to have broader mental health concepts on the CB-H (*r* = -0.14, *p* = 0.018), although no association was obtained for the CB-V (*r* = -0.05, *p* = 0.424). In contrast to Study 2, age was negatively correlated with the CB-V (*r* = -0.15, *p* = 0.009) and CB-H (*r* = -0.19, *p* = 0.001) and there were significant differences amongst the income groups on both the CB-V, *F*(3, 287) = 3.08, *p* = 0.028, and CB-H, *F*(3, 287) = 3.45, *p* = 0.017. Post hoc Tukey HSD tests and Games-Howell tests for multiple comparisons found that CB-V and CB-H scores were higher for participants with annual income < USD$50,000 than for those with income between USD$100,000 and USD$149,999.

Correlations between breadth scales and the mental health variables are presented in Table 5. The correlation between the CB-V and the CB-H scales was significantly positive, *r* = 0.52, *p* < 0.001. The CB-V scale had significant positive correlations with help-seeking attitudes and all mental health experience items, and a negative correlation with social distance. All of these correlations were small, although the correlation with personal experience of psychological problems was close to a medium effect, *r* = 0.27, *p* < 0.001. Similar or stronger correlations were obtained for the CB-H scale, with the exception of a significant negative correlation with dangerousness, *r* = -0.17, *p* = 0.004. CB-H significantly correlated with all mental health variables investigated with small to medium effects.

**Table 5:** Correlations between two concept breadth scales and other mental health variables

	2	3	4	5	6	7	8	9	10
1. Vertical breadth	.52^**^	.10	-.09	-.15^**^	.13^*^	.27^**^	.12^*^	.17^**^	.18^**^
2. Horizontal breadth		.20^**^	-.17^**^	-.21^**^	.13^*^	.32^**^	.22^**^	.23^**^	.25^**^
3. Mental health literacy			-.44^**^	-.32^**^	.26^**^	.20^**^	.23^**^	.18^**^	.26^**^
4. Dangerousness				.72^**^	-.28^**^	-.17^**^	-.14^*^	-.21^**^	-.32^**^
5. Social distance					-.26^**^	-.26^**^	-.24^**^	-.22^**^	-.31^**^
6. Help-seeking attitudes						.09	.29^**^	.19^**^	.21^**^
7. Personal experience of psychological problems							.70^**^	.49^**^	.45^**^
8. Personal help-seeking experience								.41^**^	.35^**^
9. Family’s experience of psychological problems									.49^**^
10. Friends’ experience of psychological problems									

Pearson correlations were computed for correlations amongst variables 1–5; Point-Biserial correlations were computed for correlations involving at least one variable 6–9

^*^p < .05

^**^p < .01

#### Predictive validity

To determine whether the demonstrated bivariate associations between concept breadth and stigma (perceived dangerousness and social distance), help-seeking attitudes, and personal experience variables were independent of mental health literacy, we conducted a series of regression analyses (Table 6) and logistic regression analyses for the dichotomous personal experience measures (Table 7) with each concept breadth scale and mental health literacy as predictors. All models were significant and mental health literacy was associated with lower levels of stigma, more positive help-seeking attitudes, and having personal experience of psychological problems and help-seeking. The concept breadth scales did not independently predict perceived dangerousness or help-seeking attitudes but they both predicted lower levels of social distance. They also predicted having personal experience of psychological problems, and the CB-H scale additionally predicted greater personal experience of help-seeking.

**Table 6:** Summary of regression analyses with concept breadth and mental health literacy predicting stigma and help-seeking outcomes

Outcome	MHL B	*p*	CB-V B	*p*	CB-H B	*p*	Model *R*^2^	*p*
Dangerousness	-0.14	< .001	-0.01	.406	-	-	.195	< .001
Dangerousness	-0.14	< .001	-	-	-0.01	.134	.199	< .001
Social distance	-0.08	< .001	-0.01	.031	-	-	.116	< .001
Social distance	-0.07	< .001	-	-	-0.01	.009	.122	< .001
Help-seeking	0.90	< .001	0.19	.051	-	-	.080	< .001
Help-seeking	0.88	< .001	-	-	0.08	.144	.074	< .001

*MHL* Mental health literacy

**Table 7:** Summary of logistic regression analyses with concept breadth and mental health literacy predicting personal mental health experience outcomes

Outcome	MHL B	*p*	CB-V B	*p*	CB-H B	*p*	Model *R*^2^	*p*
Personal experience	.13	.002	.09	< .001	-	-	.140	< .001
Personal experience	.11	.013	-	-	.06	< .001	.166	< .001
Personal help-seeking	.16	< .001	.03	.076	-	-	.087	< .001
Personal help-seeking	.15	< .001	-	-	.04	.002	.115	< .001

*MHL* Mental health literacy

### Discussion

Study 3 extended the previous study by establishing additional associations between the concept breadth measures and other mental health-related variables. Study 3 revealed that concept breadth has modest but consistent associations with measures of stigma, help-seeking attitudes, and personal experiences of mental health problems and seeking help for such problems. Holding broad concepts of mental disorder appears to be associated with desirable attitudes toward people experiencing mental health problems and willingness to seek professional help for these problems. It also appears to be associated with greater personal experience with mental ill health. This pattern of associations points to the promise of concept breadth as a factor to consider in understanding, studying, and potentially reducing undesirable mental health-related attitudes. If holding broad or inclusive concepts of mental disorder is correlated with more favourable attitudes, it is possible that promoting such concepts might boost those attitudes. Although evidence for that speculation awaits studies that move beyond cross-sectional correlations, it opens a new avenue in stigma and help-seeking research.

The Study 3 finding that several associations between concept breadth and other mental health variables are independent of mental health literacy is also important. Mental health literacy is a well-researched construct that is known to be associated with a range of psychological outcomes. Greater knowledge about mental health and illness is associated with lower stigma and greater help-seeking. Our finding that concept breadth continues to predict these variables, and personal experiences, even when mental health literacy is statistically controlled implies that it is capturing a factor that is implicated in mental health-related attitudes and experiences but is distinct from accurate knowledge. Having a more inclusive concept of mental disorder, regardless of the objective accuracy of that concept, may be an important factor in how people think about and respond to it. In addition, consistent findings from Study 2 and 3 that concept breadth has no associations with gender and education level provide further support that it is a distinct concept from other mental health variables that are often associated with gender and education. The consistent finding across Study 2 and 3 that liberals tend to have broader concepts of mental disorder than conservatives may help to explain the political differences in support for public mental health initiatives.

## General discussion

The studies reported here developed and validated new self-report measures of mental disorder concept breadth. This construct resonates with extensive theoretical literature on mental health and psychiatric classification, arising in relation to concerns about diagnostic inflation and “psychiatrization” [15], but it had yet to be assessed as an individual difference variable. In addition to enabling empirical research on variations in concept breadth and their implications, the CB-H and CB-V embody an important distinction between two different sources of variability, identified as horizontal and vertical breadth. The scales therefore allow individual and group differences in mental disorder concept breadth to be evaluated in a differentiated way.

Our studies support the reliability, validity, and promise of the new scales. Their internal consistency was good to very good, their unifactorial structures were supported by exploratory and confirmatory analyses, and they were found to correlate moderately without being redundant, implying that the scales capture unique variance in two forms of concept breadth. The scales converged as predicted with an existing generalized measure of harm concept breadth. They also diverged substantially from two measures of mental health literacy and did not correlate with education levels, supporting the theoretical claim that holding broad concepts of disorder is not merely a sign of more accurate and extensive knowledge of mental health. The CB-H and CB-V correlated negatively with measures of stigma and positively with measures of help-seeking attitudes and personal experience of mental ill health, and several of these associations were independent of mental health literacy, supporting the scales’ incremental validity. In sum, we believe the new scales have demonstrated solid psychometric credentials and the potential to illuminate attitudes, beliefs, and behaviors related to mental health.

The existence of reliable and validated measures of mental disorder concept breadth affords a wide range of research opportunities. Several avenues for future research could examine the correlates, determinants, and consequences of holding broad concepts of disorder. We sketch out some of these future research directions below.

With regard to correlates, it will be important to continue the construct validation of mental disorder concept breadth by exploring its associations with other individual difference variables, including personality traits, attitudes, values, and ideologies. Previous research on generalized harm-related concept breadth has found it to be associated with individual differences in empathy, liberal political orientation, justice sensitivity, endorsement of harm-based morality, and other constructs [27, 28]. It remains to be seen whether these associations hold for mental disorder-related concept breadth, although Study 2 and 3 found evidence for a positive association between CB-H and liberal political orientation.

Correlations between demographic variables and mental health concept breadth also require further exploration. Previous research has typically found greater harm-related concept breadth among women than men, along with mixed evidence for greater breadth among younger participants [27], but the present research found no gender differences on the new scales and a weak age effect only in Study 3. Given the widespread interest in shifting attitudes towards and rising prevalence of mental ill health, the possibility of age effects, even if they are weak or subtle, is important to investigate.

With regard to determinants, it is important to discover whether particular personal experiences, social environments, or cultural backgrounds influence the breadth of people’s concepts of mental disorder. It is possible that direct or indirect personal experiences of mental ill health may broaden the concept, a possibility raised by Study 3’s finding of a correlation between these variables. However, that correlation allows no causal inference, and the causal arrow could even be reversed as broad concepts might lead people to identify their problems as disorders. Cultural influences may also be important; for instance, Tse and Haslam [34] have found preliminary evidence of narrower concept breadth among Asian Americans relative to their White peers. Such cultural differences, which have received very little empirical attention to date, may have implications for ethnic disparities in stigma and help-seeking, and the new scales provide a means to study them.

The new scales could be employed as outcome measures in experimental studies of experiences or interventions that might broaden or narrow people’s disorder concepts. Work by Foulkes and Andrews [14], for example, speculates that awareness campaigns may inadvertently increase rates of mental disorder, and one mechanism through which they might do so is by vertically inflating (i.e., lowering the threshold of) people’s disorder concepts. Exposure to formal education about mental health or to mental health awareness campaigns may broaden people’s concepts of mental disorder. Although the minimal correlation between mental disorder concept breadth and mental health literacy obtained in Studies 2 and 3 implies that concept breadth should not be confused with greater or more accurate knowledge, mental health literacy could still be a possible mechanism for influencing concept breadth or vice versa. Understanding the differing correlates, determinants, and mechanisms of mental disorder concept breadth and mental health literacy is a priority for future research.

On the subject of consequences, the new scales could be used to examine the possible effects of mental disorder concept breadth on other mental health-related phenomena. Study 3’s findings suggest that holding broader concepts is beneficial for improving attitudes and promoting help-seeking, but whether concept breadth plays a causal role and the mechanisms through which it might do so remain to be established. Broader concepts may reduce stigma by supporting the view that mental disorder is common and on a continuum with normality and it may increase help-seeking by the same mechanism or by increasing the likelihood that people believe they have a disorder. Equally, broad disorder concepts may have less beneficial consequences. Consistent with Foulkes and Andrews’ [14] argument, broad concepts might dispose people to make false positive self-diagnoses, which may have problematic implications via self-fulfilling prophecy effects. These possibilities await future research, which the new scales might enable.

While this series of studies established and illustrated the new concept breadth scales and their associations, these studies were not without limitations. Although care was taken to create a diverse set of vignettes for the purposes of scale construction, that set was inevitably incomplete. Although the short form of the vertical breadth scale (CB-V-S) demonstrated a unidimensional structure, good internal consistency, and strong convergence with the CB-V, further research using the CB-V-S as a standalone measure is needed to validate it. The cross-sectional design of the studies, particularly in Study 3, did not allow causal inferences about links between concept breadth and other variables to be made. While concept breadth was shown to significantly predict social distance and personal experience of psychological problems and help-seeking, it was likely that the relationships between these variables are reversed in direction or even more likely to be bidirectional. Future experimental or longitudinal studies utilising these scales could help to clarify the nature of these associations.

## Conclusions

The CB-H and CB-V scales offer researchers an opportunity to explore new questions in mental health research. Debates about the boundaries of the concept of mental disorder have primarily been abstract and philosophical to date, but the scales provide a way to study variations in the placement of these boundaries between individuals and groups. At a time when concerns over diagnostic inflation, the psychiatrization of everyday life problems, and the rising prevalence of mental ill health are urgent, we believe it is important to investigate the causes, correlates, and consequences of the breadth of people’s concepts of mental disorder. Mental disorder concept breadth is a construct that complements existing research on mental health literacy and may offer new insights into laypeople’s mental health-related attitudes, beliefs, and behaviors.

### Supplementary Information

**Additional file 1: Appendix A.** Concept Breadth of mental disorder – Vertical scale (CB-V). **Appendix B**. Concept Breadth of mental disorder – Vertical scale – Short form (CB-V-S). **Appendix C.** Concept Breadth of mental disorder – Horizontal scale (CB-H)

## Data Availability

The dataset analysed in the current study is available from the corresponding author on reasonable request.
