# Supplementary material for: Individual differences in the expansiveness of mental disorder concepts: development and validation of concept breadth scales
Source: BMC Psychiatry. 2023 Oct 4;23:718. doi: 10.1186/s12888-023-05152-6 (PMC10548567; doi:10.1186/s12888-023-05152-6)
Supplement: Supplementary file 1 — Additional file 1: Appendix A. Concept Breadth of mental disorder – Vertical scale (CB-V). Appendix B. Concept Breadth of mental disorder – Vertical scale – Short form (CB-V-S). Appendix C. Concept Breadth of mental disorder – Horizontal scale (CB-H) [file 12888_2023_5152_MOESM1_ESM.docx]

## Appendix A

## Concept Breadth of mental disorder – Vertical scale (CB-V)

The following pages each present descriptions of five people who may or may not have a mental disorder. Their experiences vary from the **most severe** (at the **top** of the page) to the**least severe** (at the **bottom** of the page).

Your task is to decide which of the five people, if any, have a mental disorder by selecting "**Yes**" or "**No**". You must make a decision about every person. 

For example, if you think **all of the people** have a mental disorder, you would rate the five descriptions (from top to bottom) Yes Yes Yes Yes Yes, as showed below.

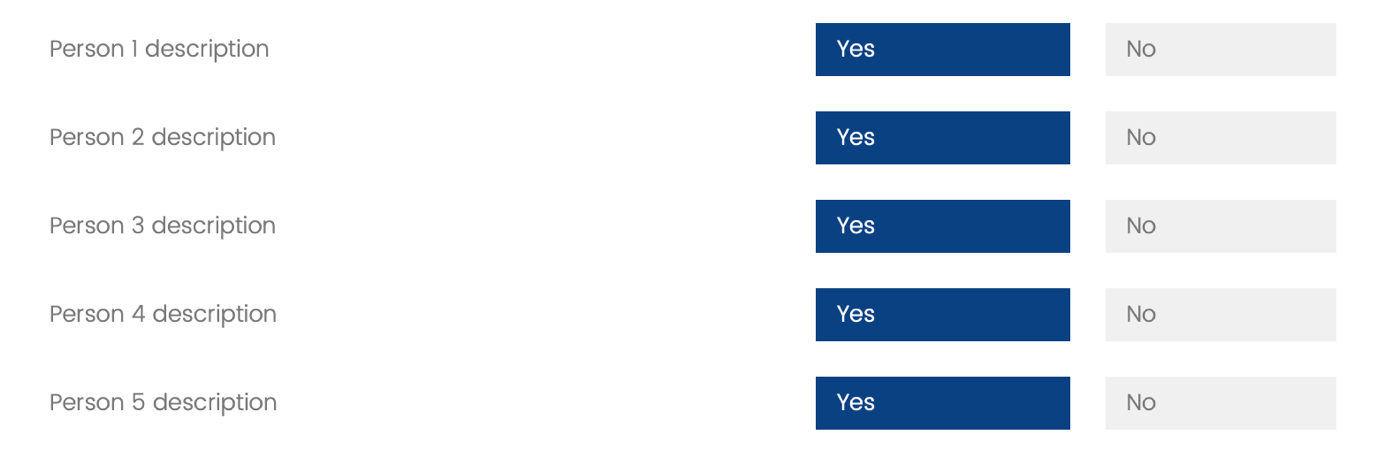


If you think only the **two most severe** people have a mental disorder, you would rate Yes Yes No No No.


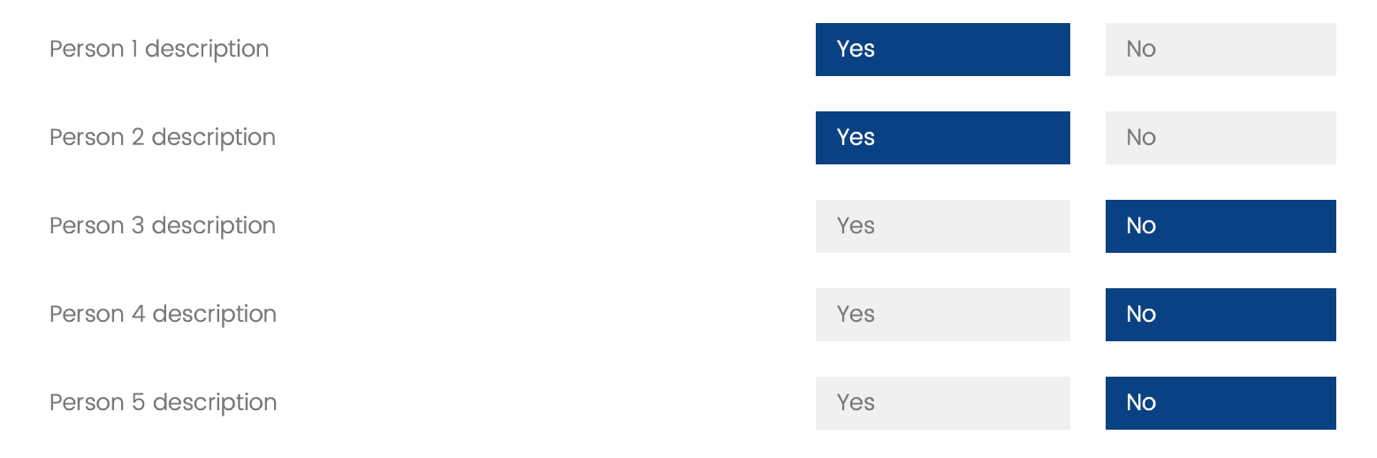


If you think the**four most** **severe** people have a mental disorder, you would rate Yes Yes Yes Yes No.

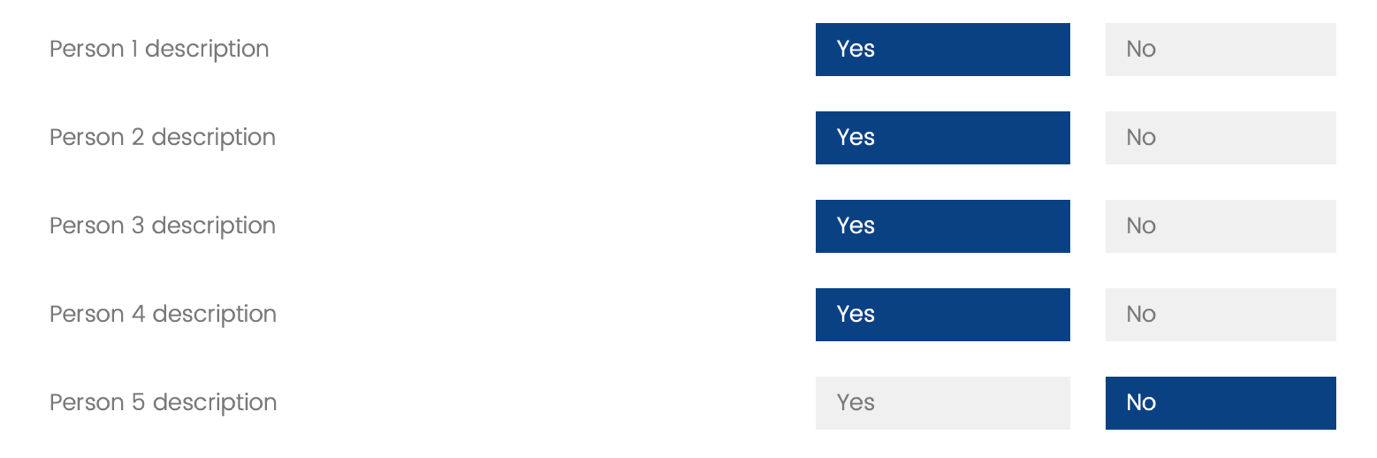


If you think**none of the people** on the page have a mental disorder, you would rate No No No No No.

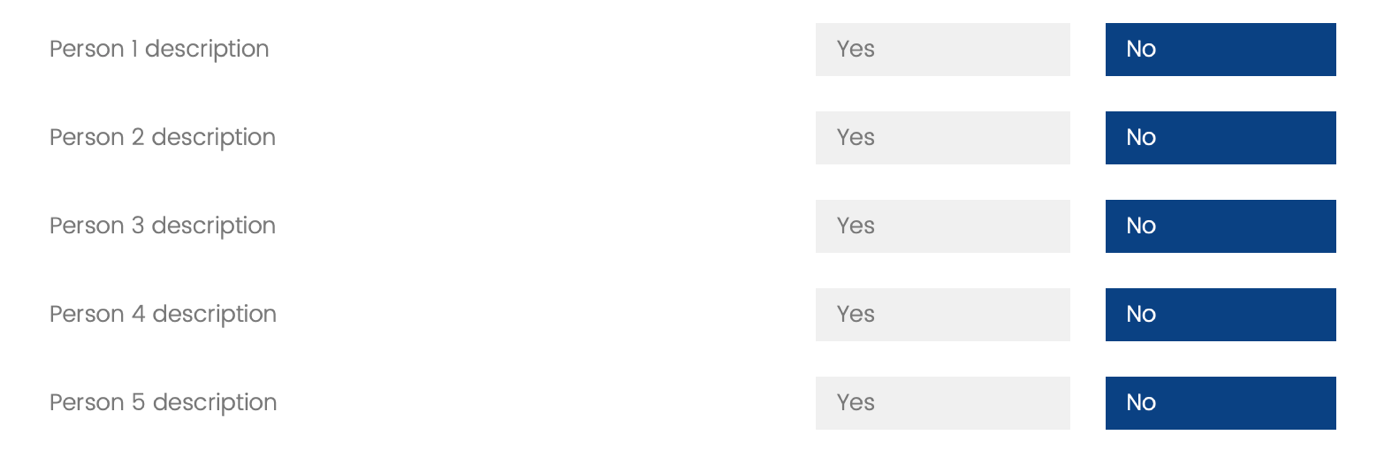


There are no right or wrong answers to this task. We are interested in your personal opinions.

To make sure that you understand the instruction, here is an **example question**.

Please read the following descriptions about five people carefully from top to bottom and make a decision about each person.

Do any of these people described below have a mental disorder?

*Imagine after reading the descriptions (there is no actual descriptions below, only illustrating the format of the questions), you think that only the person with the most severe situation has a mental disorder, please respond accordingly.*

| Person 1 description | - Yes | - No |
| --- | --- | --- |
| Person 2 description | - Yes | - No |
| Person 3 description | - Yes | - No |
| Person 4 description | - Yes | - No |
| Person 5 description | - Yes | - No |

### [Response validation: Yes, No, No, No, No]

[There are seven questions in this scale; each question starts with the introductory instructions and the question “Do any of these people described below have a mental disorder?”, each of the five vignettes in each question requires a yes or no answer*.* Question 1 below shows the full example of the instruction and question format that should be applied for all the other six questions.]

VS01_BipoI

Please read the following descriptions about five people carefully **from top to bottom**and make a decision about each person. 

**Do any of these people described below have a mental disorder?**

| This person used to be very calm and quiet, but over the past few months, they have become much more talkative and easily irritated, which has caused a lot of physical and verbal conflicts with their friends. When asked about these changes, the person explained that they feel extremely restless, that their thoughts are racing all the time and that they cannot control their thoughts and behaviors. | - Yes | - No |
| --- | --- | --- |
| This person used to be very calm and quiet, but over the past month, they have become much more talkative and easily irritated, which has caused a lot of verbal conflicts with their friends. When asked about these changes, the person explained that they feel very restless, that their thoughts are racing all the time, and that they cannot control their thoughts and behaviors. | - Yes | - No |
| This person used to be very calm and quiet, but over the past two weeks, they have become much more talkative and easily irritated, which caused a lot of conflicts with their friends. When asked about these changes, the person explained that they feel restless, that their thoughts are going faster than usual, and that they have trouble controlling their thoughts and behaviors. | - Yes | - No |
| This person is usually very calm and quiet, but over the past few days, they became much more talkative and easily irritated, which caused a lot of confusion amongst their friends. When asked about these changes, the person explained that they feel restless, that their thoughts are sometimes going faster than usual, and that they sometimes have trouble controlling their thoughts and behaviors. | - Yes | - No |
| This person is usually very calm and quiet, but for the past two days they were much more talkative and easily irritated, which surprised their friends. When asked about these changes, the person explained that they feel a little restless, that they are thinking a little faster than usual, and that they sometimes have a little trouble controlling their thoughts and behaviors. | - Yes | - No |

| VS02_MDD |
| --- |
| This person has been feeling very sad, self-critical, and upset every day for the past year. They have lost a significant amount of weight and cannot fall asleep at night, so they feel very tired all the time. They are also completely unable to concentrate and think clearly, which has caused them to lose their job. |
| This person has been feeling very sad, self-critical, and upset every day for the past two months. They have lost a lot of weight and cannot fall asleep at night, so they feel tired all the time. They are also unable to concentrate and think clearly, which has impaired their ability to work. |
| This person has been feeling very sad, self-critical, and upset every day for the past month. They have lost some weight and cannot fall asleep at night, so they feel tired all the time. They are also unable to concentrate and think clearly, which has impacted their work performance. |
| This person has been feeling very sad, self-critical, and upset every day for the past two weeks. They have lost some weight and cannot fall asleep at night, so they often feel very tired. They have trouble concentrating and thinking clearly, which has somewhat impacted their work performance. |
| This person has been feeling very sad, self-critical, and upset most days for the past two weeks. They think they have lost some weight and cannot fall asleep easily at night, so they often feel tired. Sometimes, they have trouble concentrating and thinking clearly, but it does not affect their work. |

| VS03_GAD |
| --- |
| This person is extremely anxious about every part of their life. All the time, they worry a lot about big things like getting fired, as well as little things like forgetting to bring their keys. They find it impossible to stop worrying. These anxieties constantly tense up their muscles, make them restless, and ruin their concentration. |
| This person is very anxious about almost every part of their life. Frequently throughout the day, they worry a lot about big things like getting fired, as well as little things like forgetting to bring their keys. They find it very difficult to stop worrying. These anxieties almost always tense up their muscles, make them restless, and affect their concentration. |
| This person is very anxious about many parts of their life. On and off throughout the day, they worry a lot about big things like getting fired, and sometimes about little things like forgetting to bring their keys. They find it very difficult to stop worrying. These anxieties often tense up their muscles, make them restless, and affect their concentration. |
| This person is very anxious about some parts of their life. Occasionally, they worry about big things like getting fired, and sometimes about little things like forgetting to bring their keys. They find it quite difficult but not impossible to stop worrying. These anxieties sometimes tense up their muscles and make them restless. |
| This person is anxious about specific parts of their life. Occasionally, they worry about big and small things related to their job, such as getting fired or forgetting to bring their keys. They find it quite difficult but not impossible to stop worrying. These anxieties sometimes make them a little restless. |

| VS04_OCD |
| --- |
| At least three times every day, this person has persistent thoughts that they are contaminated in some way. The only way they can make the thoughts go away is to wash their hands with soap for at least 30 minutes. If they don’t wash their hands, they feel extremely anxious. They have tried to stop their handwashing but have been completely unable to stop it. This problem led to the ending of a close relationship and makes it very difficult to hold down a job. |
| At least one time every day, this person has persistent thoughts that they are contaminated in some way. The only way they can make the thoughts go away is to wash their hands with soap for at least 15 minutes. If they don’t wash their hands, they feel very anxious. They have tried to stop their handwashing but have been unable to stop it for more than a month. This problem causes difficulties in their close relationship and makes it hard to hold down a job. |
| Most days, this person has persistent thoughts that they are contaminated in some way. The main way they can make the thoughts go away is to wash their hands with soap for at least 5 minutes. If they don’t wash their hands, they feel quite anxious. They have tried to stop their handwashing and have been able to stop doing it for up to one month. This problem causes some difficulties in their close relationship and is embarrassing at work. |
| Some days, this person thinks that they are contaminated in some way. The main way they can make the thoughts go away is to wash their hands with soap for at least 5 minutes. If they don’t wash their hands, they feel a little anxious. They have tried to stop their handwashing and can usually control it unless they are under stress. This problem causes some embarrassment with friends and at work. |
| Occasionally, this person thinks that they are unclean. One way they can make the thoughts go away is to wash their hands with soap for at least 5 minutes. If they don’t wash their hands, they don’t feel too bad, but they prefer to do it. They have tried to stop their handwashing and can usually control it. This handwashing generally doesn’t cause them many problems. |

| VS05_DID |
| --- |
| This person seems to have completely different personalities at different times. Usually, they are very shy and reserved, but sometimes they are very outspoken and confident. When they are questioned about the unusual things they have done, they have no recollection of doing them. These striking alterations in their behavior have severely affected their personal relationships and made them lose their jobs. |
| This person seems to have completely different personalities at different times. Usually, they are very shy and reserved, but sometimes they are very outspoken and confident. When they are questioned about the unusual things they have done, they claim to have no recollection of doing them. These alterations in their behavior have significantly affected their personal relationships and impaired their work performance. |
| This person seems to have different personalities at different times. Usually, they are very shy and reserved, but sometimes they are very outspoken and confident. When they are questioned about unusual things they have done, they cannot explain them. These alterations in their behavior have significantly affected their personal relationships. |
| This person seems to behave in very different ways at different times. Usually, they are shy and reserved, but sometimes they are outspoken and confident. When they are questioned about their inconsistent behavior, they cannot explain them. These alterations in their behavior have made other people have trouble trusting them. |
| This person seems to behave in different ways at different times. Sometimes, they are shy and reserved, but at other times they are outspoken and confident. When they are questioned about their inconsistent behavior, they say they are a bit temperamental. These alterations in their behavior sometimes confuse other people. |

| VS06_Binge |
| --- |
| This person eats much more than most people every day. Even when they don’t feel hungry, they eat a lot very quickly until they are uncomfortably full to the point of feeling sick. Noticing this pattern of over-eating for the past few months, they feel extremely upset about it, hate themselves, but also feel completely helpless about controlling how much they eat. |
| This person eats much more than most people several days a week. Even when they don’t feel hungry, they eat a lot very quickly until they are uncomfortably full to the point of feeling a little sick. Noticing this pattern of over-eating for the past few months, they feel very upset about it, are critical of themselves, but also feel helpless about controlling how much they eat. |
| This person eats more than most people sometimes. Even when they don’t feel hungry, they eat a lot very quickly until they are uncomfortably full. Noticing this occasional pattern of over-eating for the past few months, they feel upset about it but also feel a little helpless about controlling how much they eat. |
| This person eats more than most people occasionally. Even when they don’t feel hungry, they eat a lot very quickly until they are uncomfortably full. Noticing this occasional pattern of eating for the past few months, they feel very concerned about it but also feel it's very challenging to control how much they eat. |
| This person eats more than most people once in a while. Even when they don’t feel hungry, they eat a lot very quickly until they are uncomfortably full. Noticing this pattern of eating once in a while for the past few months, they feel concerned about it but feel it's somewhat challenging to control how much they eat. |

| VS07_AvoidPer |
| --- |
| This person is extremely afraid of disapproval and rejection and often thinks they have been rejected or judged negatively when they have not. They make up excuses so they never have to join any group activities at work. They have extremely low self-esteem and assume that all people are judgmental and critical. They are too scared to meet and talk to new people in social situations and as a result they are completely socially isolated. They rarely interact with people even if they are certain that they will be accepted unconditionally. |
| This person is very afraid of disapproval and social rejection. They make up excuses to avoid any group activities at work. They have very low self-esteem and assume that all people are judgmental and critical. They are very scared to meet and talk to new people in social situations and as a result they are socially isolated. They rarely interact with people unless they are certain that they will be accepted unconditionally. |
| This person is very afraid of disapproval and social rejection. They make up excuses to avoid group activities at work. They have low self-esteem and assume that most people are judgmental and critical. They are very reluctant to meet and talk to new people in social situations. They are unwilling to interact with people unless they are certain that they will be accepted unconditionally. |
| This person is afraid of disapproval and social rejection. Sometimes they make up excuses to avoid group activities at work. They have relatively low self-esteem and assume that most people are judgmental and critical. They are often reluctant to meet and talk to new people in social situations. They are unwilling to initiate interactions with people unless they are certain that they will be accepted unconditionally. |
| This person dislikes disapproval and social rejection. Occasionally, they make up excuses to avoid group activities at work. They have relatively low self-esteem and assume that some people are judgmental and critical. They don't want to meet and talk to new people in social situations. They are unwilling to initiate interactions with people unless they are fairly sure that they will be accepted by them. |

[Scoring: Yes = 1; No = 0. Sum up all scores, range = 0 – 35 where 35 indicates a large vertical breadth of mental disorder.]

## Appendix B

## Concept Breadth of mental disorder – Vertical scale – Short form (CB-V-S)

Please select your response to the statement “Does this person have a mental disorder?” in terms of “Yes” or “No”.

**This person has a mental disorder.**

| V1 | This person shows few emotions on their face. They are interested in some forms of social activity but prefer to sit alone sometimes for half an hour or so. They hold a few very unusual beliefs, and their speech is sometimes a little hard to follow. | - Yes | - No |
| --- | --- | --- | --- |
| V2 | This person is usually very calm and quiet, but over the past few days, they became much more talkative and easily irritated, which caused a lot of confusion amongst their friends. When asked about these changes, the person explained that they feel restless, that their thoughts are sometimes going faster than usual, and that they sometimes have trouble controlling their thoughts and behaviors. | - Yes | - No |
| V3 | This person has been feeling very sad, self-critical, and upset every day for the past two weeks. They have lost some weight and cannot fall asleep at night, so they often feel very tired. They have trouble concentrating and thinking clearly, which has somewhat impacted their work performance. | - Yes | - No |
| V4 | This person is very anxious about some parts of their life. Occasionally, they worry about big things like getting fired, and sometimes about little things like forgetting to bring their keys. They find it quite difficult but not impossible to stop worrying. These anxieties sometimes tense up their muscles and make them restless. | - Yes | - No |
| V5 | Some days, this person thinks that they are contaminated in some way. The main way they can make the thoughts go away is to wash their hands with soap for at least 5 minutes. If they don’t wash their hands, they feel a little anxious. They have tried to stop their handwashing and can usually control it unless they are under stress. This problem causes some embarrassment with friends and at work. | - Yes | - No |
| V6 | This person seems to behave in very different ways at different times. Usually, they are shy and reserved, but sometimes they are outspoken and confident. When they are questioned about their inconsistent behavior, they cannot explain them. These alterations in their behavior have made other people have trouble trusting them. | - Yes | - No |
| V7 | This person eats more than most people sometimes. Even when they don’t feel hungry, they eat a lot very quickly until they are uncomfortably full. Noticing this occasional pattern of over-eating for the past few months, they feel upset about it but also feel a little helpless about controlling how much they eat. | - Yes | - No |
| V8 | This person has been causing trouble ever since they entered high school half a year ago. They are a little cruel to animals and they sometimes bully and initiate fights with other students. They sometimes damage other people’s property but show some remorse when they do. | - Yes | - No |
| V9 | Once a month, this person lies to their family to visit a casino to play poker. Whenever they lose big money, they stay a little longer at the casino to chase their losses. Their gambling has led to minor financial losses and occasionally impacted their work performance. They tried quitting gambling once, but the success was short-lived. | - Yes | - No |
| V10 | This person is very afraid of disapproval and social rejection. They make up excuses to avoid group activities at work. They have low self-esteem and assume that most people are judgmental and critical. They are very reluctant to meet and talk to new people in social situations. They are unwilling to interact with people unless they are certain that they will be accepted unconditionally. | - Yes | - No |

[Scoring: Yes = 1; No = 0. Sum up all scores from 10 questions, range = 0 – 10 where 10 indicates a large vertical breadth of mental disorder.]

## Appendix C

## Concept Breadth of mental disorder – Horizontal scale (CB-H)

[This scale starts with the introductory instructions below, and then all 15 vignettes are followed by the statement “**This person has a mental disorder.**” And a 6-point Likert scale response, 1 = *Strongly disagree* and 6 = *Strongly agree.* Question 1 below shows the full example of the instruction and question format that should be applied for all the other 14 questions in this scale.]

HS_Intro

You will be reading a series of descriptions about some people. Please read each description and rate the extent to which you agree or disagree with the subsequent statement. We are interested in your personal views, there is no right or wrong answer.

HS01_Persist

This person has been feeling down for the past few years. They often feel a sense of hopelessness and have low self-esteem, but these feelings come and go day by day. On bad days their work suffers, but on better days they can work effectively.

**This person has a mental disorder.**

- Strongly disagree (1)
- Disagree (2)
- Somewhat disagree (3)
- Somewhat agree (4)
- Agree (5)
- Strongly agree (6)

HS02_SocAn

This person is terrified of public speaking because they are worried that they will be judged negatively by others. They fear the embarrassment if they say the wrong things. Therefore, they have been avoiding any events or occasions that require them to speak in front of people.

HS03_PTSD

This person witnessed a terrible car accident a month ago and since then they have been having nightmares about the accident every other night. The dream wakes them up in fear which keeps them awake and can sometimes last for the whole day. Although the accident and feeling are vivid in the dream, they fail to recall any specific details about the accident when asked. Because of this accident, they are easily startled by loud noises and they avoid the area where the accident had happened.

HS04_Soma ​​​​​

This person has been experiencing back pain for a long while. They are extremely worried that their back pain will lead to them being paralyzed. They have spent months consulting multiple doctors at various hospitals. All doctors have prescribed painkillers and assured them there is no risk of becoming paralyzed.

HS05_Insom

This person has had trouble sleeping since they were a child. For most nights, it takes hours for them laying on the bed before they can fall asleep. This has led to them having low energy and poor concentration for their daytime activities.

HS06_Delirium

This person has been suffering from alcohol withdrawal. In the past few days, after quitting drinking, they have had difficulty focusing and thinking clearly. They also feel disoriented and often forget things that just happened. Sometimes these confusions get very serious, and their speech can become incoherent.

HS07_Narcis

This person feels special and is very proud of their achievements. They believe that only similar high-status people are worth their time and they expect people to recognize their importance and give them special treatment. They lack sensitivity and empathy for the feelings of others, and often exploit and manipulate people around them for personal gain.

HS08_Cheat

This person has been in a long-term romantic relationship with the same partner. However, for the past few years, they have been cheating on their partner. When their partner discovered this, they begged for forgiveness, but their cheating continued soon after.

HS09_Jealousy

This person constantly suspects that their romantic partner is cheating on them. They question their partner’s whereabouts and sometimes follow them to see who they are meeting. Their partner has complained about these controlling behaviors, which often resulted in arguments. The person has tried to stop doubting their partner’s fidelity, but their doubts always come back.

HS10_Selfishness

This person always puts themself first and has no regard for other people. For example, they cut in front of other people in queues and play their music very loud late at night when others are trying to sleep. They don’t share important information with others, and they are unwilling to help others.

HS11_SocMedia

This person uses social media almost constantly. Their parents often complain about their use of social media, and this has caused a lot of serious conflicts in the past. The person has learned to lie about the amount of time they spend on social media to avoid having arguments. If their friends suggest an activity that temporarily stops them from using social media, such as playing games, they usually don’t take part in the activity.

HS12_ChronicFati

This person has been feeling extremely tired for the past few months. Even when they sleep more than 10 hours the night before, they do not feel refreshed when they get up. This has been affecting their job performance since they are unable to concentrate and stay alert for their working hours.

HS13_Gaming

This person plays a lot of online games and they have been skipping work to play games at an increasing frequency over the past year. They have lost interest in spending quality time with their partner and constantly think about games even when they are not playing. When their partner complains about this and forces them to stop playing, they become very irritable and sad for a couple of days. Repeatedly, they lie to their partner so they can play online games and this conflict eventually led the couple to separate.

HS14_Dhat

This person suffers from premature ejaculation and also thinks that they are losing semen-like white fluid when urinating. They have become very anxious and upset about this because they see semen as a “vital fluid” and think losing it equates to a loss of health. They also feel a loss of appetite and energy.

HS15_Imposter

This person is a high achiever, but they constantly feel that their achievements have been based on pure luck. They doubt their competence and fear that they cannot live up to people’s expectations of them. They always work very hard to make sure that no one will find out that they are a fraud.

[Scoring: Sum up all scores from 15 questions, range = 15 – 90 where 90 indicates a large horizontal breadth of mental disorder.]
